# Loss of MAGEC3 Expression Is Associated with Prognosis in Advanced Ovarian Cancers

**DOI:** 10.3390/cancers14030731

**Published:** 2022-01-30

**Authors:** James Ellegate, Michalis Mastri, Emily Isenhart, John J. Krolewski, Gurkamal Chatta, Eric Kauffman, Melissa Moffitt, Kevin H. Eng

**Affiliations:** 1 Department of Cancer Genetics and Genomics, Roswell Park Comprehensive Cancer Center, Buffalo, NY 14263, USA; James.Ellegate@RoswellPark.org (J.E.J.); Michalis.Mastri@RoswellPark.org (M.M.); Emily.Isenhart@RoswellPark.org (E.I.); John.Krolewski@RoswellPark.org (J.J.K.); 2 Department of Medicine, Roswell Park Comprehensive Cancer Center, Buffalo, NY 14263, USA; Gurkamal.Chatta@RoswellPark.org; 3 Department of Urology, Roswell Park Comprehensive Cancer Center, Buffalo, NY 14263, USA; Eric.Kauffman@RoswellPark.org; 4 Department of Gynecologic Oncology, Roswell Park Comprehensive Cancer Center, Buffalo, NY 14263, USA; Melissa.Moffitt@RoswellPark.org; 5 Department of Biostatistics and Bioinformatics, Roswell Park Comprehensive Cancer Center, Buffalo, NY 14263, USA

**Keywords:** MAGEC3, ovarian cancer, prognostic biomarker, machine learning

## Abstract

**Simple Summary:**

Familial studies connect variants in the X-linked gene MAGEC3 to early-onset ovarian cancers. In this retrospective cohort study, we determined that, unlike other MAGE family members, the MAGEC3 protein is normally expressed in ovarian tissue but is lost in half of the ovarian cancers. Similar to other predisposition genes like BRCA2, survival modeling suggests that expression loss is associated with favorable progression-free survival, and continued expression is associated with response to platinum therapy. Because of the assumed antigenicity of MAGE genes, we tested and observed associations with lymphocyte infiltration, NY-ESO-1 seropositivity, and the co-expression of tumor antigens at Xq28. Using transcriptomic modeling, we predicted that MAGEC3 expression is associated with stress-related cell cycle stalling and DNA repair pathway expression.

**Abstract:**

Rare variants in MAGEC3 are associated with BRCA negative, early-onset ovarian cancers. Given this association, we evaluated the impact of MAGEC3 protein expression on prognosis and transcription. We quantified normal and tumor protein expression of MAGEC3 via immunohistochemistry in *n* = 394 advanced ovarian cancers, assessed the correlation of these values with clinicopathologic and immunological features and modeled survival using univariate and multivariate models. To extend these results, we quantified MAGEC3 protein expression in *n* = 180 cancers and used matching RNA sequencing data to determine MAGEC3-associated differentially expressed genes and to build an RNA-based model of MAGEC3 protein levels. This model was tested in a third independent cohort of patients from TCGA’s OV dataset (*n* = 282). MAGEC3 protein was sporadically lost in ovarian cancers, with half of the cases falling below the 9.5th percentile of normal tissue expression. Cases with MAGEC3 loss demonstrated better progression-free survival [HR = 0.71, *p* = 0.004], and analyses performed on predicted protein scores were consistent [HR = 0.57 *p* = 0.002]. MAGEC3 protein was correlated with CD8 protein expression [Pearson’s r = 0.176, *p* = 0.011], NY-ESO-1 seropositivity, and mRNA expression of tumor antigens at Xq28. Results of gene set enrichment analysis showed that genes associated with MAGEC3 protein expression cluster around G2/M checkpoint (NES = 3.20, FDR < 0.001) and DNA repair (NES = 2.28, FDR < 0.001) hallmark pathways. These results show that MAGEC3 is a prognostic biomarker in ovarian cancer.

## 1. Introduction

We previously reported the linkage between early-onset, BRCA negative ovarian cancers and a locus on Xq27.2 that contains the gene MAGEC3 [1] identified from inheritance patterns in the Familial Ovarian Cancer Registry (Buffalo, NY, USA) [2] that heavily favored clustering among sisters likely due to a father who transmits the risk allele [3]. MAGEC3 belongs to the melanoma antigen gene (MAGE) family whose members are defined by a common 200 residue protein domain (protein family 01454) [4] conserved from a single copy in fungi with heavy duplication in mammals and primate-specific clades [5]. Members of the family have varied reports of function in DNA repair, cell cycle control, and protein interaction [6], especially in the context of cancer [7], as well as potential use as stemness markers [8]. Historically, the MAGE family has been studied as bona fide tumor-specific antigens, and we have previously reported on their individual and combined effects on ovarian cancer prognosis [9,10]. While tumor expression of most “Type I” tumor-specific MAGE genes are associated with poor prognosis, one-third of MAGE family members are “Type II” MAGE genes that are constitutively expressed and perform essential tasks in many tissues. The level of tissue-specific expression and clinical implications of MAGEC3 expression are currently unknown. In this work, we measured MAGEC3 protein levels in ovarian cancers and normal tissues. Additionally, we measured MAGEC3 levels in samples of various tissue types containing matched RNA sequencing data which were used to build a robust predictor of protein expression. Clinical correlations and molecular associations related to MAGEC3 expression (or loss of expression) elucidate MAGE biology.

## 2. Materials and Methods

### 2.1. Patient Cohorts and TMA Construction

Three cohorts of patients were used in this study: (1) a “discovery cohort” of ovarian cancer patients from Roswell Park, (2) a “model-building” cohort of mutually exclusive patients from Roswell Park that had been accrued and molecularly characterized through the TCGA project and (3) a validation cohort of TCGA patients from other institutions without protein data.

With respect to the first cohort, following review by the Roswell Park Institutional Review Board, we obtained clinical data and archival samples for women with a primary diagnosis of ovarian cancer treated by maximal debulking surgery and first-line platinum-based chemotherapy. We required that patients had available tumor blocks taken prior to initiating systemic therapy. In total, there were *n* = 411 ovarian cancer patients (high-grade serous ovarian cancer, *n* = 333) for analysis. Tissue blocks were organized into tissue microarrays as previously described (three representative cores from each tumor) [9] and stained for MAGEC3 protein expression. TMAs contained reference cores for normalization across different blocks. TMAs also contained cores of normal tissues, which were quantified for analysis.

### 2.2. Antibody Validation

The MAGEC3 antibody was vetted by the Human Protein Atlas [11] (HPA052067) following the guidelines of the International Working Group for Antibody Validation [12] and was scored as approved for IHC and supported for antigen specificity. The antigen binds both dominant isoforms of MAGEC3 (ESNP000000386566 and ENSP00000440444) outside of the functional MAGE homology domain.

### 2.3. Immunohistochemistry

Formalin-fixed paraffin-embedded sections were cut at 4 µm, placed on charged slides, and dried at 60 °C for one hour. Slides were cooled to room temperature and added to the Dako Omnis autostainer, where they were deparaffinized with Clearify (catalog# CACLEGAL, American Mastertech, Lodi, CA, USA) and rinsed in water. Flex TRS High pH (catalog# GV804, Dako, Santa Clara, CA, USA) was used for target retrieval for 60 min. Slides were incubated with MAGEC3 antibody (catalog# HPA052067, Sigma Aldrich, Saint Louis, MO, USA) for 50 min at 1:50 dilution. Flex Rabbit Linker (catalog# GV80911-2, Dako, Santa Clara, CA, USA) was applied for 10 min followed by Flex/HRP polymer (catalog# DM843, Dako, Santa Clara, CA, USA), which was applied for 20 min followed by DAB (Diaminobenzidine) (catalog# K3468, Dako, Santa Clara, CA, USA) for 5 min for visualization. Slides were counterstained with Hematoxylin for 8 min then put into water. After removing slides from the Omnis, they are dehydrated, cleared, and cover slipped. Cores were averaged by patient and checked for outliers, and slides were normalized to common internal control spots included in the design of the TMA. MAGEC3 was quantified using an IHC Profiler [13]. CD8 (catalog# M7103, Dako, Santa Clara, CA, USA) was stained as previously described [14]. MAGEA3, MAGEA4, and MAGEA10 were stained as previously described [9] and scored as positive or negative. PRAME was stained and quantitatively scored as previously described [15]. After staining, slide-specific staining intensity and rank and file associations were tested to identify any slide/batch or spatial effects.

### 2.4. TCGA Study Data

We downloaded clinical and expression data for the TCGA Pan-Cancer (PANCAN) cohort directly from the UCSC Xena browser as batch effect normalized mRNA data (log2 RSEM, upper-quartile normalized [16]). Modifications made to the data after download include the removal of unknown or hypothetical transcripts in the Entrez database and the setting of NA counts to 0. Corresponding clinical information for the expression data were obtained from PANCAN’s curated clinical data [17].

### 2.5. Statistical Methods

Statistical tests are described as they are used in the text. In general, Student’s *t*-test, chi-square test, and log-rank test were employed as appropriate for continuous, categorical, or survival time data. All statistical tests were two-sided, and *p* < 0.05 was considered significant throughout. Survival analysis by Cox’s proportional hazards model used analysis of deviance-based model selection and employed residual tests for non-proportionality [18]. Overall survival was defined as a cancer-associated death event censored by the end of follow-up. For ovarian cancers, progression-free survival was defined as doubling of baseline CA125 confirmed with CT scan or the initiation of relapse chemotherapy regimens as defined in [19]. All analyses were performed in the R statistical programming language version 3.6.1 (R Foundation for Statistical Computing, Vienna, Austria).

### 2.6. LASSO Model Building

Within the model-building cohort, genes with consistent univariate association were screened for MAGEC3 association, and the top 1000 features with univariate association with MAGEC3 [20] were selected for multivariate model building. We used a LASSO-penalized linear model objective to select and estimate coefficients and selected the tuning parameter by unbiased leave-out-one (LOO) cross-validation via R/glmnet [21]. Suboptimal local solutions were ruled out by multiple randomized partitions of the model/validation cohorts.

DEG calling was performed in R/limma using the moderated t-statistics with FDR control [22]. The linear model was stratified on the TCGA study. Gene set analysis was performed using the R/fgsea package [23]. Annotations were maintained via R/org.Hs.eg.db and MSigDb’s Hallmark pathway set. Pathways with fewer than 15 represented genes were removed from consideration.

## 3. Results

### 3.1. MAGEC3 Protein Normal Tissue Expression and Cancer Tissue Expression

**Figure 1:**
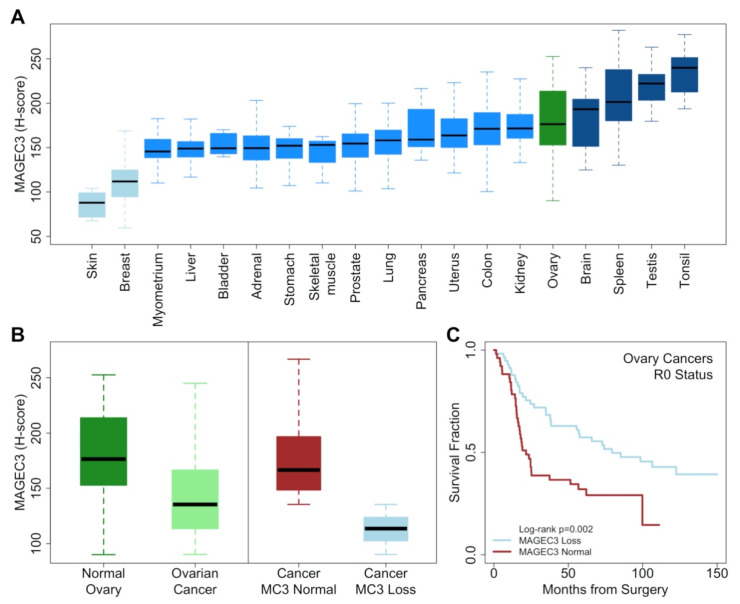
MAGEC3 protein expression in normal and cancer tissues. (**A**) MAGEC3 protein expression in normal tissue cores. (**B**) (Left side) MAGEC3 expression in normal ovary tissue and ovarian cancer. (Right side) Ovarian cancer expression levels were dichotomized at the median into “Normal” (red) and “Loss” (blue). (**C**) Kaplan–Meier plot showing progression-free survival trends for ovarian cancer patients with optimal cytoreduction stratified by MAGEC3 level.

In normal tissues, MAGEC3 was widely expressed at a similar level except for decreased expression in breast and skin tissues and increased expression in tonsil, brain, and testis tissues (Figure 1A). Normal ovary tissues had a mean expression of 181.2 on a 0–300 point scale (H-score, median 176.5, sd 40.8) while unmatched ovarian cancers had significantly lower expression (mean 145.2, median 135.4, sd 40.4; two-sample *t*-test, *p* < 0.001) (Figure 1B).

Stratified at the median of tumor expression (corresponding to the 9.5th percentile of normal ovary expression), higher levels of MAGEC3 were not different from the normal ovary (*t*-test *p* = 0.368), while lower levels of MAGEC3 were significantly lower (*p* < 0.001), reflecting loss of expression (Figure 1B). We subsequently named these tumor groups “Normal” and “Loss”. Normal MAGEC3 levels were associated with cases ascertained after 2006 (OR = 5.86, 95%CI: 3.8–9.3, *p* < 0.001) (Table 1).

### 3.2. MAGEC3 Association with Prognosis in Epithelial Ovarian Cancer

In ovarian cancers with survival follow-up (*n* = 394), normal MAGEC3 levels were associated with complete response to maximal debulking surgery followed by platinum-based chemotherapy (CR mean 150.5 versus 138.9, two-sample *t*-test, *p* = 0.008). Following the initial complete response, women who were platinum-sensitive (more than 9 months of progression-free survival after the end of platinum chemotherapy) continued to show higher MAGEC3 levels (platinum-sensitive mean 149.8 versus 140.2, *t*-test *p* = 0.033). The inverse was seen for progression-free survival (PFS) in all women with epithelial ovarian cancer (normal MAGEC3: 16.2 months median versus 24.1 months, log-rank *p* = 0.002) (Table 1). Overall survival (OS) was significantly lower in women with normal MAGEC3 and optimal cytoreduction (R0, 64.7 months versus 118.6, *p* = 0.050) but not in women with suboptimal cytoreduction (not R0, 33.9 versus 34.4, *p* = 0.900).

The age at diagnosis, FIGO stage, grade, and R0 cytoreduction were univariately associated with prognosis in ovarian cancers (Table 2). MAGEC3 level was independently associated with PFS (HR = 1.41, 95%CI:1.14–1.75, *p* = 0.002) (Figure 1C). Stepwise multivariate model selection by analysis of deviance led to a model that predicted progression-free survival using FIGO Stage (IIIC/IV, HR = 3.14, score test *p* < 0.001), histological grade (Poorly/Undifferentiated, HR = 1.38, *p* = 0.017), cytoreduction status (not R0, HR = 1.45, *p* = 0.022) and MAGEC3 level (Normal, HR = 1.41, *p* = 0.004) stratified on histological type and decade of life at diagnosis (global proportional hazards test *p* = 0.32). Women were followed for an average of 52.6 months (maximum 244.4 months). These results were not affected by the year of ascertainment effect (PFS log-rank *p* = 0.40, OS, *p* = 0.20).

### 3.3. MAGEC3 Is Associated with CD8+ T Cell Infiltrated Tumors

Given that ovarian cancers are highly immunogenic, we considered the association between MAGEC3 and the count of CD8+ tumor-infiltrating lymphocytes (TILs). We previously reported CD8+ TIL counts stained in ovarian cancer slides from these TMAs [14]. CD8+ TIL levels were positively correlated with MAGEC3 expression in ovarian tumors (Pearson’s *r* = 0.176, *p* = 0.011) (Figure S1A).

### 3.4. Relationship with Other CT Antigens and Seropositivity

We have previously reported on the expression of MAGE-A3, MAGE-A4, MAGE-A10, and PRAME [9,15] in a subset of these ovarian cancers. Among all complete records (*n* = 72), MAGEC3 was not strongly correlated with any MAGE-A antigens (A3, *r* = 0.02, *p* = 0.870; A4, *r* = 0.16, *p* = 0.185; A10, *r* = −0.06, *p* = 0.604) (Figure S2A–C). A larger set of patients (*n* = 195) had complete information on MAGEC3, PRAME (an autosomal antigen) [15] and NY-ESO-1 (gene CTAG1B at Xq28) expression and serology [10]. MAGEC3 was strongly correlated with PRAME expression (*r* = 0.422, *p* < 0.001, adjusted for year of diagnosis) (Figure S2D) and uncorrelated with NY-ESO-1 expression (*r* = 0.101, *p* = 0.157) (Figure S2E). Conversely, patients who were seropositive for NY-ESO-1 had upregulated expression of MAGEC3 (H-score β = 11.2, *p* = 0.0323) (Figure S2F). Logistic regression predicted NY-ESO-1 seropositivity was specific to MAGEC3 (+10 H-score units, OR = 1.26, *p* = 0.035) versus PRAME (OR = 1.16, *p* = 0.233).

### 3.5. MAGEC3 Protein Expression across Multiple Disease Sites

FFPE blocks were available from cases submitted by Roswell Park to the Cancer Genome Atlas (TCGA) project from several tissue sites (*n* = 180 tumors as a TMA). We stained these cores and analyzed their correlation with bulk mRNA sequencing data. Due to limited sample sizes within tissue sites, we considered a limited correlative analysis of protein expression in this set of patients (Table S1). As there is a known association between MAGEC3 and prognosis in ovarian cancer, we stratified common primary disease sites of the TCGA cohort by MAGEC3 expression. Only colorectal sites had an association with MAGEC3, displaying normal expression more frequently than MAGEC3 loss (*p* = 0.04). When considering the whole cohort, CD8+ cell levels also have a notable association with MAGEC3 expression (CD8+ cell values associated with normal MAGEC3 expression) (*p* < 0.001).

### 3.6. Modeling MAGEC3 Protein via mRNA

Much like other MAGE genes and X-linked cancer-testis antigens, MAGEC3 mRNA from bulk sequencing had a low dynamic range despite measurable protein expression and demonstrated some linear correlation with protein expression (Pearson’s *r* = 0.267, *p* = 0.0003), likely due to a few high leverage points (Spearman’s ρ = −0.003, *p* = 0.968). This observation suggested that, while some high expressing cases could be detected, RNA sequencing lacks the sensitivity to detect MAGEC3 mRNA levels.

**Figure 2:**
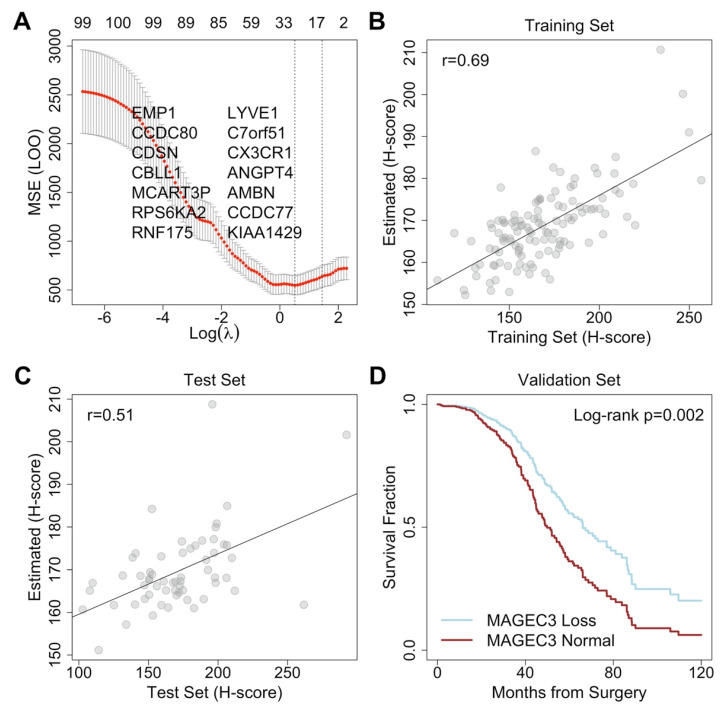
An RNA-based predictive model of MAGEC3 protein expression. (**A**) Leave out one (LOO) cross-validation error for tuning parameter selection. Fitted values in the (**B**) training set and (**C**) withheld test set. (**D**) Survival estimate validation in the independent pan-TCGA dataset for ovarian cancer.

To maximize the utility of mRNA datasets, we modeled protein expression of MAGEC3 using other mRNA measurements and divided the *n* = 180 labeled TCGA set (Table S1) into training and validation datasets (2:1 ratio) where the TCGA ovary (OV) [24] dataset was considered an unlabeled validation cohort. The final model selected by the 1 standard error (se) rule had a training mean square error (MSE) of 455.5 (Figure 2A) and a leave out one test MSE of 637.9 (*n* = 120, se: 110.11). The fully withheld validation set MSE was 885.3 (*n* = 60). Correlation between true MAGEC3 protein scores and predicted scores was 0.69 (*p* < 0.001) in the model building cohort and 0.51 (*p* < 0.001) in the validation cohort (Figure 2B,C). Similar error rates and correlations, such as those identified within these cohorts, imply a well-fit model with predictive modeling capabilities. The fitted model selected mRNA expression of AMBN, CCDC77, RNF175, C7orf51, MCART3P, CBLL1, and KIAA1429 as positively correlated with MAGEC3 protein and RPS6KA2, LYVE1, CCDC80, CX3CR1, EMP1, CDSN, and ANGPT4 as negatively correlated with MAGEC3 protein.

### 3.7. MAGEC3 Protein Predictor in Pan-TCGA Study

We applied the MAGEC3 protein level predictor to an independent set of cases sequenced and processed for TCGA’s pan-cancer analysis (*n* = 282 ovarian cancer patients), which did not have measured MAGEC3 protein levels. Analyses performed on our discovery cohorts were also performed on these predicted MAGEC3 scores. As previously observed, progression-free survival had a significant negative relationship with normal MAGEC3 scores (HR = 1.35 *p* = 0.048), which persisted after accounting for age, stage, and initial response to chemotherapy (HR = 1.74 *p* = 0.002). When split at the median, higher MAGEC3 cases had a median survival of 36.9 months (95% interval: 34.8–43.7) versus 51.3 months (45.0–62.1) (Figure 2D).

### 3.8. MAGEC3 Expression Is Associated with Stress-Related Processes

**Figure 3:**
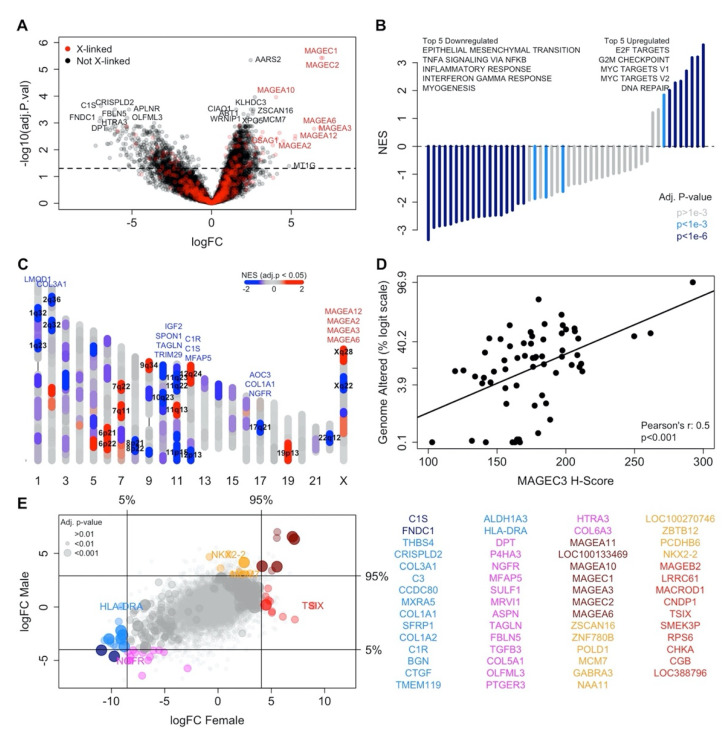
MAGEC3 protein DEG and GSEA analyses and association with genomic instability. (**A**) Volcano plot showing genes that are differentially expressed with continuous MAGEC3 protein expression. (**B**) Gene sets that are significantly associated with continuous MAGEC3 protein expression. (**C**) Ideogram showing where significantly expressed genes are located within the genome. Genes that were individually significant are listed above their locus and loci that are GSEA significant (FDR ≤ 0.05) are labeled. (**D**) Scatterplot showing the correlation between fraction genome altered and MAGEC3 protein levels in patients with PTEN, FAT4, BRAF, PTPRT, NF1, RB1, ATM, ATRX, IDH1, and TP63 mutations. (**E**) Comparison of DEGs between male and female patients.

Following the creation of the predictor, we used the mRNA data to determine the impact of MAGEC3 protein on gene expression. MAGEC3-associated differentially expressed genes (DEGs, FDR ≤ 0.5) adjusted for tissue site were more frequently upregulated across this TCGA subset (Figure 3A). We observed highly upregulated levels for the rest of the MAGEC locus (MAGEC1, and MAGEC2 on Xq27.2) and the neighboring MAGEA locus (MAGEA2, MAGEA3, MAGEA6, MAGEA10, and MAGEA12 on Xq28). Among these MAGE proteins, MAGEA2 and MAGEC2 interact with TRIM28 affecting the cell cycle among other processes [25,26]. MAGEC3 also upregulates a stress-related gene, WRNIP1 [27], a DNA replication gene, MCM7 [28], and cancer-related genes, XPO5 [29] and ZSCAN16 [30]. In addition, MAGEC3 downregulates genes that are shown to induce tumor progression, including APLMR [31,32], FBLN5 [33], and FNDC1 [34,35]. Gene set enrichment analysis (GSEA) showed that MAGEC3 significantly enriched E2F targets (NES = 3.63, FDR < 0.001), G2/M checkpoint (NES = 3.20, FDR < 0.001), and DNA repair (NES = 2.28, FDR < 0.001), whereas the epithelial to mesenchymal transition was downregulated (NES = −3.34, FDR < 0.001) (Figure 3B). We also observed that differentially expressed genes were clustered in specific genomic locations such as 11p15, 11p23, 12p13, 17q21, and Xq28 (Figure 3C). These data suggest that MAGEC3 may induce a transcriptional response that involves the expression of stress-related genes that affect the cell cycle and DNA repair while downregulating genes involved in tumor progression.

### 3.9. MAGEC3 Expression Is Correlated with Genomic Instability in Patients with Mutated Oncogenes

Due to MAGEC3’s association with specific expression patterns in various genomic locations and upregulation of DNA repair processes, we investigated the association of MAGEC3 with genomic instability. We analyzed tumor mutation burden (TMB) data from 63 patients within the TCGA patient cohort stratified on the most mutated genes. A positive correlation between levels of MAGEC3 protein and fraction genome altered (Pearson’s *r* = 0.5, *p* < 0.001) was observed in patients with PTEN, FAT4, BRAF, PTPRT, NF1, RB1, ATM, ATRX, IDH1, and TP63 mutations (Figure 3D). This association was negligible in patients without these oncogenic mutations (Pearson’s *r* = 0.03, *p* < 0.001) (Figure S3).

### 3.10. MAGEC3 Expression Associates with Different Genes Depending on Sex

Stratifying by sex, we determined which DEGs were male or female-specific or common to both groups (Figure 3E). Of particular interest was TSIX, a long non-coding RNA that inhibits XIST expression, thereby preventing X chromosome inactivation [36]. We observed a large log-fold change of TSIX expression in female patients with MAGEC3 protein expression indicative of a link between MAGEC3 protein expression and general X-chromosome inactivation. Another female-specific DEG is the MHC class II gene HLA-DRA, which was specifically downregulated [37]. Male-specific DEGs include MCM7, a DNA replication licensing factor [28]; NKX2-2, an important biomarker for metastatic prostate cancers [38]; and NGFR, the canonical single-pass transmembrane receptor linked to melanoma [39] and squamous cell carcinoma [40].

## 4. Discussion

To our knowledge, this is the first investigation of the primate-specific MAGEC3 protein expression and its relation to cancer. MAGEC3 appears to be expressed in multiple tissues, including the tonsil, spleen, brain, and testes, at a similar level. Normal ovary tissues had a relatively high expression, while MAGEC3 levels were unusually low in breast and skin tissues. This makes MAGEC3 similar to Type II MAGE family members with ubiquitous expression and to the updated classification, “Type 1c: not restricted” [41].

Consistent with the supposition that it is a tumor suppressor, MAGEC3 levels were lower on average in ovarian cancers than in normal ovary tissue. Other reports studying MAGEC3 mRNA (using Affymetrix oligonucleotide arrays, probe 216592_at) show associations with positive prognosis in hepatocellular carcinoma [42] and mixed relapse-free survival based on lymph node status in breast cancer [43].

We reported that MAGEC3 was difficult to measure by bulk RNA sequencing and subsequently developed an RNA-based linear model to predict MAGEC3 protein levels learned from labeled data. The accuracy of the predictor was evaluated in a validation set and subsequently applied to ovarian cancer patients within the TCGA pan-cancer dataset. Using the predicted scores, we validated the results found for cases with directly measured MAGEC3 scoring. The ability to accurately predict protein levels based on an RNA predictor greatly increased the sample size and impact of our results.

We observed that specific genes associated with MAGEC3 protein expression were clustered around G2/M checkpoint and DNA repair hallmark pathways, which is consistent with reports that other MAGE family members impact the cell cycle (MAGEC2) [44], damage sensing via p53 targeting E3-ligases (MAGEC2) [45] or ATM/ATR phosphorylation (MAGED2) [46], and genome stability (MAGEG1) [47], all of which are associated with stress response [6]. An interesting conjecture emerges that these non-MAGE-driven, high MAGEC3 expressing tumors may retain their MAGE-related putative DNA repair functions. In addition, differentially expressed genes associated with MAGEC3 were clustered in specific genome locations indicating that MAGEC3 may affect chromatin accessibility due to a stress-related program [48] that can be related to cell cycle and DNA repair.

Tumor mutational burden is often correlated with proteins associated with DNA damage repair [49]. MAGEC3 protein levels were positively associated with fraction genome altered in patients with PTEN, FAT4, BRAF, PTPRT, NF1, RB1, ATM, ATRX, IDH1, and TP63 mutations. Because this strong correlation with fraction genome altered was only observed in patients with these driver mutations, we believe that expression of MAGEC3 is secondary to the mutations. The tumors may be expressing MAGEC3 in response to high genomic instability caused by their driver mutations.

MAGE proteins are cancer-testis antigens [50]. MAGEC3 protein was associated with increased expression of mRNA in the MAGE-C locus (at Xq27) and the MAGE-A locus (at Xq28). Interestingly, MAGEC3 was a good predictor for seropositive ovarian cancer patients even though NY-ESO-1 (at Xq28) mRNA and protein were not associated with MAGEC3 expression. These results, along with the correlation of MAGEC3 protein and CD8 positivity, indicate that MAGEC3 may be related to the re-opening of Xq28 and the expression of tumor antigens.

While more work is required to assess its operating characteristics, higher relative MAGEC3 tumor protein was a significant biomarker for poor prognosis in ovarian cancer. Specifically, cases with loss of MAGEC3 expression had an unusually good prognosis. We interpret this as evidence that MAGEC3-driven disease etiology is driven by the loss of MAGEC3’s protective expression. The favorable prognosis of these cases is consistent with the observation that women with germline BRCA1 and BRCA2 loss of function mutations have a favorable [51] but transient [52] response to platinum-based chemotherapy. We also observed that MAGEC3 is associated with initial platinum response despite the fact that MAGEC3 loss patients have better survival trends after the termination of treatment. This suggests that MAGEC3 has distinct roles depending on platinum treatment status and that active platinum treatment elicits a specific response from MAGEC3. This would also explain why MAGEC3 shows an opposite correlation with survival in hepatocellular carcinoma [28], which is not predominantly treated with platinum.

In contrast, those cases with higher MAGEC3 expression and poor prognosis likely reflect a MAGE-independent form of the disease (and not a pro-tumor function for MAGEC3) because the level of tumor expression is not significantly increased above normal tissue. The association with other X-chromosome antigens may point to a general mechanism of immunogenic activation; the re-activation of MAGEC3 in these cases may be due to a bystander effect of this process.

A limitation of our investigation involves the use of retrospective cohorts. We did observe a tissue processing effect for FFPE blocks obtained prior to 2006 that did not significantly affect our conclusions after stratification and sensitivity analysis. The use of immunohistochemistry to read the MAGE protein levels is semi-quantitative, and slide-to-slide variation was minimized using normal controls on each tissue microarray and automatic staining in an experienced core pathology facility. This protocol was used in previous work with other stains. The MAGEC3 antibody was vetted by the Human Protein Atlas [11] and proven to be appropriate for IHC.

Despite these limitations, our study used a large single-institution cohort of patients with definitive power to provide the first investigation of MAGEC3 protein expression in ovarian cancer cases and to confirm the gene’s relevance to cancer biology and patient prognosis. In an independent, model-building cohort and a further validation cohort, MAGEC3 was identified as a candidate prognostic biomarker associated with ovarian cancer with the potential for predictive marker-based treatment strategies based on targeting cases with high expression.

## 5. Conclusions

MAGEC3 is a prognostic biomarker in ovarian cancer. Specifically, loss of MAGEC3 protein expression is associated with favorable progression-free survival. We propose that the mechanism of MAGEC3-disease driven etiology is related to its antigenicity and/or association with stress-related cell cycle stalling and DNA repair pathway expression. Further studies are needed to confirm these hypotheses and advise treatment approaches for non-loss cases.

## Figures and Tables

**Table 1:** Discovery cohort clinical characteristics. Clinical characteristics of the ovarian cancer discovery cohort by MAGEC3 protein level.

Characteristic	All Ovary Patients	MAGEC3 Loss	MAGEC3 Normal	*p*-Value
	*n* = 411	*n* = 206	*n* = 205	
Age of Diagnosis (years)				0.3 ^†^
Mean (range)	63 (21–93)	63 (21–93)	64 (21–89)	
Missing	0	0	0	
Year of Diagnosis (*n* (%))				<0.001 ^‡^
Before 2006	226 (57.7)	149 (78.4)	77 (38.1)	
After 2006	166 (42.3)	41 (21.6)	125 (61.9)	
Missing	19	16	3	
Primary (*n* (%))				0.1 ^‡^
Ovary	339 (83.5)	177 (86.8)	162 (80.2)	
Primary peritoneal	67 (16.5)	27 (13.2)	40 (19.8)	
Missing	5	2	3	
FIGO Stage (*n* (%))				0.1 ^‡^
I/II/IIIA/B	70 (17.4)	41 (20.4)	29 (14.4)	
IIIC/IV	333 (82.6)	160 (79.6)	173 (85.6)	
Missing	8	5	3	
Grade (*n* (%))				0.3 ^‡^
Well/Moderately differentiated	108 (26.7)	59 (29.4)	49 (24.1)	
Poorly/Undifferentiated	296 (73.3)	142 (70.6)	154 (75.9)	
Missing	7	5	2	
Histology (*n* (%))				0.2 ^‡^
Serous	333 (81.0)	161 (78.2)	172 (83.9)	
Other Epithelial	78 (19.0)	45 (21.8)	33 (16.1)	
Missing	0	0	0	
Cytoreduction (*n* (%))				0.6 ^‡^
R0	109 (26.8)	57 (28.2)	52 (25.5)	
Not R0	297 (73.2)	145 (71.8)	152 (74.5)	
Missing	5	4	1	
Treatment Outcome (*n* (%))				0.06 ^‡^
Complete response	197 (56.8)	90 (51.4)	107 (62.2)	
Not complete response	150 (43.2)	85 (48.6)	65 (37.8)	
Missing	64	31	33	
Platinum Sensitivity (*n* (%))				0.4 ^‡^
Sensitive	170 (53.6)	83 (51.2)	87 (56.1)	
Resistant	147 (46.4)	79 (48.8)	68 (43.9)	
Missing	94	44	50	
Survival (months)				
Median progression-free survival	18.9	24.1	16.2	0.002 ^§^
Median overall survival	43.0	45.8	40.2	0.2 ^§^
^†^ *p*-value was calculated using Student’s *t*-test.		*n* may vary by characteristic due to missing data.		
^‡^ *p*-value was calculated using the chi-squared test.				
^§^ *p*-value was calculated using the log-rank test.				

**Table 2:** Discovery cohort survival analysis. Univariate and multivariate survival analyses of epithelial ovarian cancer (global proportional hazards test *p* = 0.32).

Ovarian Cancer		Univariate Analysis (*n* = 411) ^†^			Multivariate Analysis (*n* = 394)		
Covariate	Risk Level	Hazard Ratio	95% CI	*p*-Value ^‡^	Hazard Ratio	95% CI	*p*-Value ^‡^
Age	+10 years	1.17	(1.08–1.27)	<0.001		Stratifier ^§^	
Stage	I/II/IIIA/B		Reference			Reference	
	IIIC/IV	4.09	(2.83–5.93)	<0.001	3.14	(1.93–5.08)	<0.001
Grade	Well/Moderately differentiated		Reference			Reference	
	Poorly/Undifferentiated	1.29	(1.01–1.65)	0.04	1.38	(1.06–1.79)	0.02
Histology	Other Epithelial		Reference			Stratifier ^§^	
	Serous	1.19	(0.89–1.58)	0.2			
Cytoreduction	R0		Reference			Reference	
	Not R0	2.37	(1.81–3.10)	<0.001	1.45	(1.05–1.99)	0.02
MAGEC3 Level	Loss		Reference			Reference	
	Normal	1.41	(1.14–1.75)	0.002	1.41	(1.12–1.79)	0.004
^†^ *p*-value was calculated using Student’s *t*-test.			*n* may vary by characteristic due to missing data.				
^‡^ *p*-value was calculated using the chi-squared test.							
^§^ *p*-value was calculated using the log-rank test.							

## Data Availability

The data and code used in this manuscript are available upon request. Publicly available TCGA datasets used in this manuscript were downloaded and can be found through UCSC Xena Browser (https://xenabrowser.net/datapages/) (accessed on 21 March 2020).

## Supplementary Materials

The following supporting information can be downloaded at: https://www.mdpi.com/article/10.3390/cancers14030731/s1, Figure S1: MAGEC3 expression correlates with CD8 expression, Figure S2: MAGEC3 association with MAGE-A antigens and seropositivity, Figure S3: MAGEC3 is not correlated with tumor mutation burden in tumors lacking driver mutations, Table S1: TCGA validation cohort characteristics.
