# Supplementary material for: Loss of MAGEC3 Expression Is Associated with Prognosis in Advanced Ovarian Cancers
Source: Cancers (Basel). 2022 Jan 30;14(3):731. doi: 10.3390/cancers14030731 (PMC8833712; doi:10.3390/cancers14030731)
Supplement: Supplementary file 1 [file cancers-14-00731-s001.zip › cancers-1555409-supplementary.pdf]

cancers

MDPI

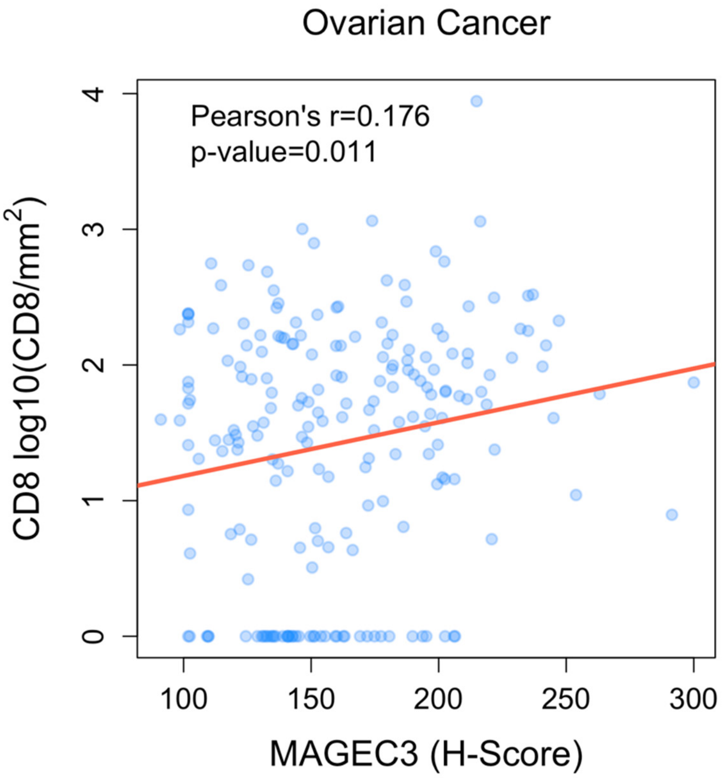

Figure S1. MAGEC3 expression correlates with CD8 expression. TMAs were stained and scored to determine protein levels of MAGEC3 (H-score) and CD8 (stain intensity per mm^2). Plotting those levels reveals a positive correlation between MAGEC3 and CD8 in ovarian cancer cases.

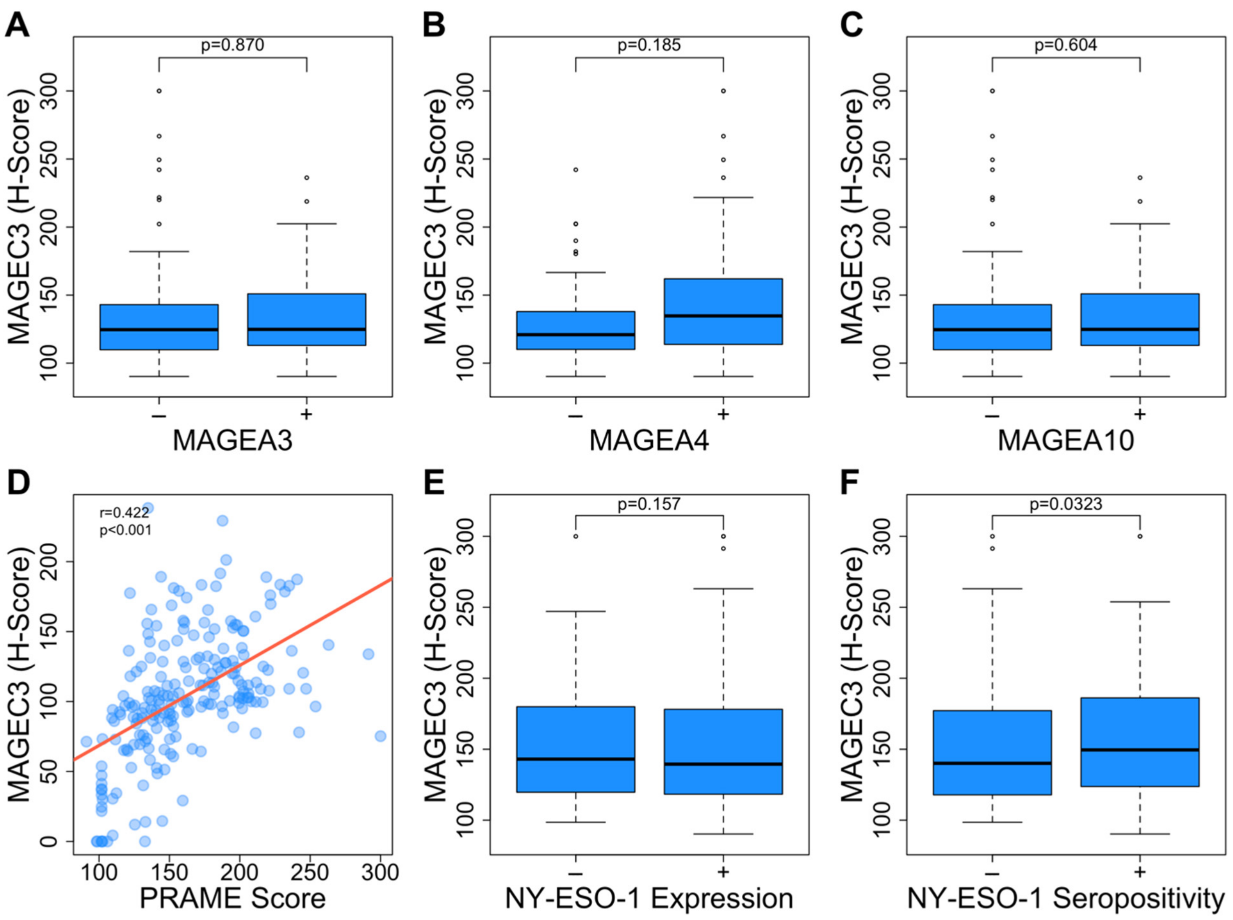

Figure S2. MAGEC3 association with MAGE-A antigens and seropositivity. A subset of 72 ovarian cancer patients within our discovery cohort contained binary expression data for MAGE-A antigens. There is no significant correlation between MAGEC3 and (A) MAGEA3, (B) MAGEA4, or (C) MAGEA10. A slightly larger set of 195 ovarian cancer patients had data for PRAME expression, NY-ESO-1 expression, and NY-ESO-1 seropositivity. There is a positive correlation between MAGEC3 and (D) PRAME levels as well as (F) NY-ESO-1 seropositivity, but not between MAGEC3 and (E) NY-ESO-1 expression.

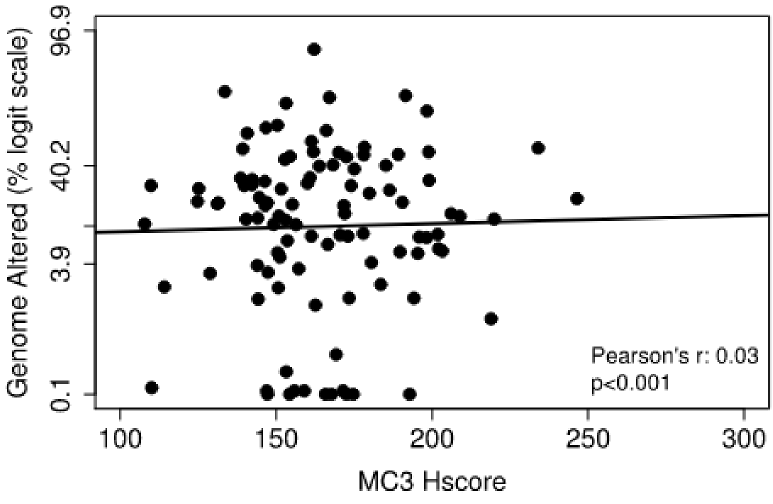

Figure S3. MAGEC3 is not correlated with tumor mutation burden in tumors lacking driver mutations. Scatterplot showing that fraction genome altered is not correlated with MAGEC3 protein levels in patients lacking *PTEN*, *FAT4*, *BRAF*, *PTPRT*, *NF1*, *RB1*, *ATM*, *ATRX*, *IDH1*, or *TP63* mutations.

Table S1. TCGA validation cohort characteristics. Clinical characteristics of the TCGA validation cohort by MAGEC3 protein level.

Characteristic	All Patients	MAGEC3 Loss	MAGEC3 Normal	P-value
	n=180	n=90	n=90	
**Age of Diagnosis [years]**				
Mean (range)	60 (24-90)	60 (24-90)	60 (30-90)	0.97†
Missing	1	1	0	
**Year of Diagnosis [n (%)]**				
Before 2006	49 (27.4)	29 (32.6)	20 (22.2)	0.17‡
After 2006	130 (72.6)	60 (67.4)	70 (77.8)	
Missing	1	1	0	
**Primary Site* [n (%)]**				
Colorectal	28 (23.0)	10 (16.7)	18 (29.0)	0.04‡
Kidney	17 (13.9)	11 (18.3)	6 (9.7)	0.23‡
Breast	14 (11.5)	11 (18.3)	3 (4.8)	0.10‡
Lung	14 (11.5)	9 (15.0)	5 (8.1)	0.61‡
Sarcomas	7 (5.7)	1 (1.7)	6 (9.7)	0.82‡
Prostate	6 (4.9)	5 (8.3)	1 (1.6)	0.30‡
Ovary	5 (4.1)	1 (1.7)	4 (6.5)	0.27‡
Melanoma	5 (4.1)	2 (3.3)	3 (4.8)	1.00‡
Thyroid	5 (4.1)	4 (6.7)	1 (1.6)	0.47‡
Other	21 (17.2)	6 (10.0)	15 (24.2)	
Missing	58	30	28	
**Sex [n (%)]**				
Male	102 (57.0)	48 (53.9)	54 (60.0)	0.50‡
Female	77 (43.0)	41 (46.1)	36 (40.0)	
Missing	1	1	0	
**Race [n (%)]**				
Black or African American	23 (13.8)	14 (17.1)	9 (10.6)	0.32‡
White	142 (85.0)	67 (81.7)	75 (88.2)	
Other	2 (1.2)	1 (1.2)	1 (1.2)	
Missing	13	8	5	
**AJCC Tumor Stage [n (%)]**				
I	28 (29.5)	18 (35.3)	10 (22.7)	
II	25 (26.3)	12 (23.5)	13 (29.6)	0.66‡
III	27 (28.4)	10 (19.6)	17 (38.6)	
IV	15 (15.8)	11 (21.6)	4 (9.1)	
Missing	85	39	46	
**CD8 Level [% positive area]**				
Mean (range)	36 (11-77)	30 (11-60)	42 (22-77)	< 0.001†
Missing	34	17	17	
**Survival [months]**				
Median progression-free survival	72.0	84.3	71.5	0.62§
Median overall survival	117.9	NA	117.9	0.51§

†P-value was calculated using Student's t-test.
‡P-value was calculated using the chi-squared test.
§P-value was calculated using the log-rank test.
NA-median not achieved
*Rare sites: lymph node, uterus, endometrium, urinary bladder, adrenal gland, liver, omentum, abdominal wall, cervix, mesentery, myometrium, pancreas, spleen, stomach, thymus

n may vary by characteristic due to missing data
